# Household transmission of SARS-CoV-2 in the paediatric age group and associated factors: an ambispective community-based study

**DOI:** 10.3389/fpubh.2026.1778406

**Published:** 2026-04-08

**Authors:** Aslısu Akdeniz Vurunbigi, Ibrahim Etem Piskin

**Affiliations:** Department of Pediatrics, Faculty of Medicine, Zonguldak Bulent Ecevit University, Zonguldak, Türkiye

**Keywords:** household transmission, paediatric COVID-19, post-COVID, reinfection, SARS-CoV-2, secondary attack rate

## Abstract

**Background:**

Households represent a major setting for SARS-CoV-2 transmission due to prolonged close contact and shared living environments. Although household transmission has been widely investigated, studies focusing on households including children and incorporating long-term follow-up data remain limited.

**Methods:**

This ambispective community-based observational study included 119 households comprising 501 individuals in Zonguldak, Türkiye, all containing at least one child and at least one laboratory-confirmed COVID-19 case between March 2020 and March 2021. Retrospective data were used to assess intra-household transmission and calculate the household secondary attack rate (SAR). Subsequently, households were followed prospectively for approximately 2 years (until March 2023) to evaluate reinfection, post-COVID symptoms, vaccination status, and mortality. Household-level and individual-level factors associated with transmission were analysed.

**Results:**

Intra-household transmission occurred in 63.0% of households, and complete household infection was observed in 23.5% of households. The mean household secondary attack rate was 40.7%. Adult age of the index case, presence of comorbidities, symptomatic disease, and close-contact behavior’s such as shared sleeping arrangements and shared meals were associated with increased transmission risk. In contrast, implementation of household isolation and mask use significantly reduced the likelihood of complete household infection. During long-term follow-up, reinfection occurred in 8.4% of participants, post-COVID symptoms were reported in 27.1% of confirmed cases, and mortality was observed only among older individuals with multiple comorbidities.

**Conclusion:**

This ambispective household-based study with extended follow-up demonstrates that SARS-CoV-2 transmission in families with children is common and strongly influenced by modifiable behavioural factors. Household-level preventive measures, particularly effective isolation and mask use, may play a crucial role in reducing transmission and mitigating long-term consequences of COVID-19 in paediatric-inclusive households.

## 1 Introduction

Severe Acute Respiratory Syndrome Coronavirus 2 (SARS-CoV-2) infection has posed a major global public health challenge since late 2019 and was rapidly declared a pandemic. The primary modes of transmission include respiratory droplets and aerosols, with the risk of transmission increasing substantially in enclosed and crowded settings (1, 2). In this context, households play a critical role in the transmission of SARS-CoV-2, as they constitute living environments in which prolonged and close contact is unavoidable. Numerous studies conducted during the pandemic have demonstrated that the household secondary attack rate is higher than that observed in community settings (3).

The clinical course of Coronavirus Disease 2019 (COVID-19) varies substantially according to age. In the paediatric population, the disease frequently presents as asymptomatic or mild, which has led to the role of children in transmission chains being overlooked for a prolonged period. Consequently, early studies predominantly focused on adult populations, and the contribution and susceptibility of children to household transmission were not adequately evaluated. Nevertheless, the impact of factors such as symptom presence, disease severity, comorbidities, intra-household behaviours, shared space utilisation, and adherence to preventive measures on household transmission dynamics remains unclear (4).

As the pandemic progressed, the emergence of reinfections, widespread vaccination, and post-COVID symptoms demonstrated that COVID-19 is not merely an acute infection, but a disease with substantial long-term individual and societal consequences (1). However, long-term, household-based follow-up data remain limited.

The aim of this study was to calculate the household secondary attack rate (SAR) in households with confirmed COVID-19, to identify individual, environmental, and behavioural factors influencing intra-household transmission of SARS-CoV-2, and to evaluate the role of children in household transmission dynamics. In addition, the study sought to elucidate factors associated with the infectivity of index cases and the susceptibility of household contacts.

Furthermore, long-term follow-up data were analyzed to assess determinants associated with reinfection, development of post-COVID symptoms, and mortality.

## 2 Materials and methods

### 2.1 Study design

This study was designed as a community-based, ambispective observational–analytical investigation incorporating both retrospective and prospective components, with the aim of evaluating intra-household SARS-CoV-2 transmission dynamics. In the retrospective component, index cases with confirmed COVID-19 and their household contacts residing in the same household were identified between 12 March 2020 and 1 March 2021. In the prospective component, these households were followed longitudinally, and epidemiological and clinical outcomes were systematically assessed.

The primary objective of the study was to determine SARS-CoV-2 transmission from index cases to household contacts and to estimate the household secondary attack rate (SAR). Secondary analyses focused on long-term follow-up outcomes, including COVID-19 vaccination status, occurrence of new infection and/or reinfection, development of post-COVID symptoms, and mortality.

### 2.2 Study setting and period

The study was conducted in Zonguldak province, Türkiye, under the coordination of Zonguldak Bülent Ecevit University Faculty of Medicine, Training and Research Hospital. Households were identified between 12 March 2020 and 1 March 2021, and individuals included in the study were followed prospectively for approximately 2 years, until 1 March 2023. Data obtained during the retrospective period were derived from existing medical records, whereas data collected during the prospective follow-up were obtained through active surveillance and structured interviews.

### 2.3 Study population, inclusion criteria, and sampling

The study population comprised households residing in Zonguldak province, Türkiye, consisting of at least two individuals and including at least one child aged under 18 years. The study sample was drawn from households in which at least one individual had a confirmed diagnosis of COVID-19 based on reverse transcription–polymerase chain reaction (RT-PCR) positivity and/or compatible thoracic computed tomography findings.

Owing to isolation measures implemented during the pandemic, limitations in field access, and structural constraints related to contact tracing, eligible households were identified through index cases diagnosed at the university hospital and subsequently included in the study.

Households were eligible for inclusion if they met the following criteria:

1) Presence of at least one individual with a confirmed diagnosis of COVID-19 within the household;
2) Presence of at least one household contact in addition to the index case;
3) Presence of at least one paediatric individual (<18 years), including the index case if applicable.

To identify eligible households, 202 households were contacted by telephone and assessed for eligibility through index cases diagnosed with COVID-19 between 1 March 2020 and 1 March 2021. Among these households, 119 agreed to participate and met the inclusion criteria, and were therefore included in the study.

### 2.4 Sample size

The sample size was calculated based on the highest reported mean secondary attack rate (SAR) of 30%, identified in meta-analyses examining intra-household SARS-CoV-2 transmission during 2020–2021.

Assuming a 95% confidence level, 5% margin of error, and a design effect of 1.5, the minimum required sample size was estimated to be 100 households. Accordingly, 119 households, comprising 501 individuals, were included in the study.

### 2.5 Data collection and follow-up

Study data were collected between 12 March 2020 and 1 March 2023 through face-to-face interviews and telephone interviews conducted by trained researchers using a pre-designed structured questionnaire.

The structured questionnaire included standardized items designed to assess household preventive behaviours and close-contact practices during the acute infection period following identification of the index case. These variables included mask use within the household, adherence to home isolation of the index case, and contact-related behaviours such as sharing meals, sleeping in the same room, or sleeping in the same bed with the index case.

Information was obtained through self-report from adult household members, while data regarding paediatric participants were provided by parents or legal guardians. Participants were asked to recall household behaviours occurring during the acute infection period following identification of the index case. Preventive behaviours were recorded as categorical variables (e.g., mask use within the household: yes/no; adherence to home isolation: yes/no; shared meals: yes/no).

Following the initial assessment, participants were followed up by telephone at predefined time points, including day 7, day 15, day 30, month 3, month 6, year 1, and year 2. Importantly, no households were lost during the follow-up period, and all 119 households (501 individuals) completed the study.

During the early phase of the COVID-19 pandemic, testing capacity was limited and routine RT-PCR testing of asymptomatic household contacts was not systematically implemented. Consequently, some household contacts were not tested. In the statistical analyses evaluating SARS-CoV-2 transmission, Asymptomatic household contacts who did not undergo diagnostic testing during the acute infection period were analysed together with PCR-negative individuals in the primary transmission analyses. This approach reflects testing practices during the early pandemic period and has been used in several epidemiological studies investigating household transmission.

**Figure 1:**
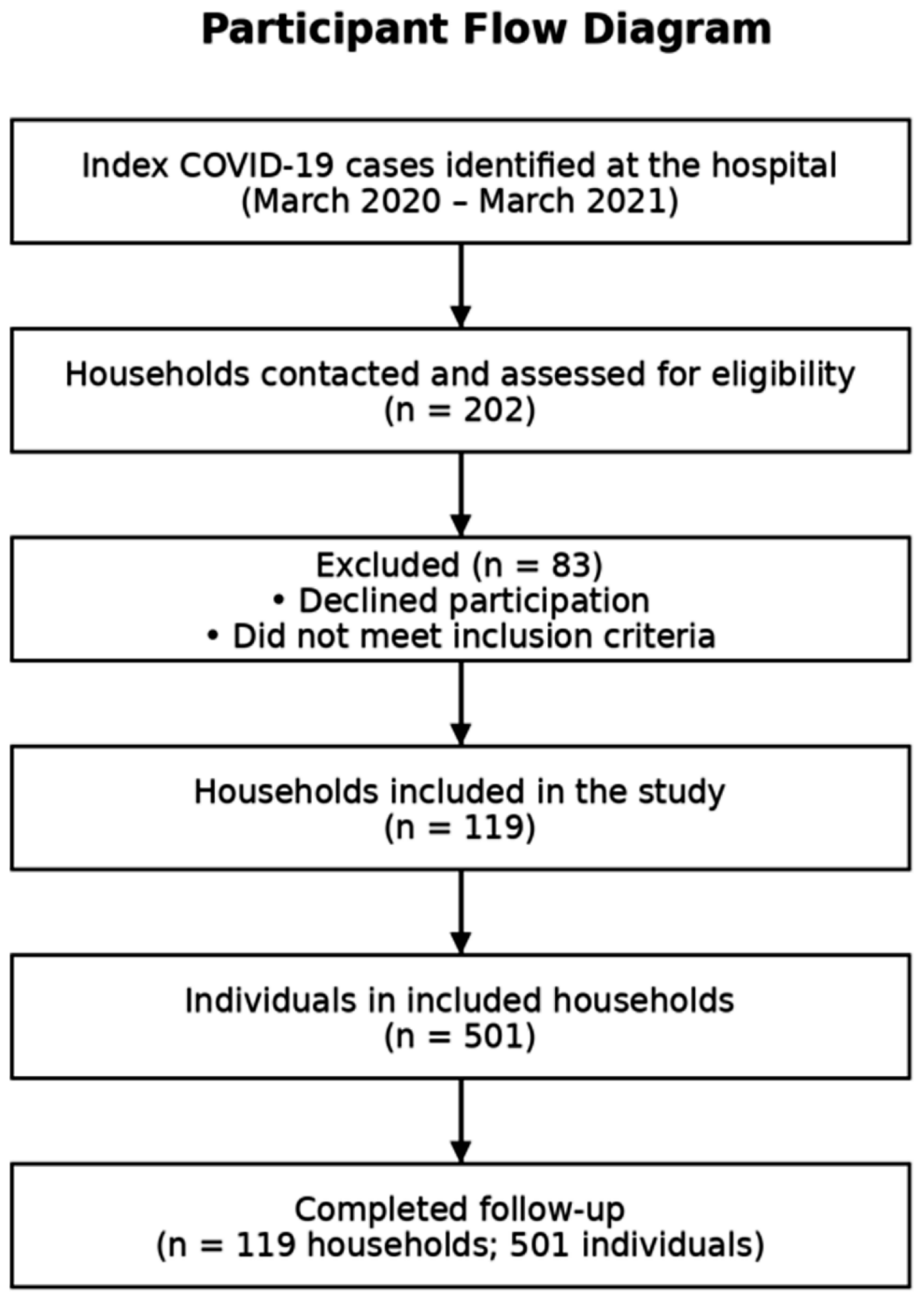
Flow diagram of household recruitment and participant inclusion. The flow diagram illustrates the process of household identification, eligibility assessment, and inclusion in the study. A total of 202 households were contacted and screened for eligibility. Among them, 83 households either declined participation or did not meet the inclusion criteria. Finally, 119 households comprising 501 individuals were included in the study and completed the follow-up period. No households were lost during follow-up.

The participant recruitment process is illustrated in Figure 1.

### 2.6 Case definitions: index case, secondary case, and COVID-19 diagnosis

The index case was defined as the individual within the household who first developed symptoms and/or was the first to have laboratory-confirmed SARS-CoV-2 infection.

Secondary cases were defined as household contacts who developed symptoms and/or received a diagnosis of COVID-19 at least 48 h after the index case and within 21 days of symptom onset in the index case. In households in which the index case was asymptomatic, this interval was calculated from the date of diagnosis.

The 21-day transmission window was selected based on published epidemiological studies of household SARS-CoV-2 transmission and the known incubation period of COVID-19, which typically ranges between 2 and 14 days, but may extend beyond this period in household exposure settings.

In households where two or more individuals developed symptoms or received a diagnosis within a short time interval and it was not possible to clearly determine the first infected individual, these individuals were considered co-primary cases. In such situations, the individual with the earliest reported symptom onset was designated as the index case for analytical purposes. When the temporal order could not be clearly established, those cases were excluded from analyses requiring strict index–secondary classification.

A diagnosis of COVID-19 was confirmed based on the presence of at least one of the following criteria:

- Positive RT-PCR test for SARS-CoV-2
- Radiological findings on thoracic computed tomography consistent with COVID-19 pneumonia

### 2.7 Secondary attack rate

The secondary attack rate (SAR) was calculated using the following formula:

SAR (%) = [(total number of confirmed cases within the household—index case)/(total number of household members—index case)] × 100.

All SAR calculations were performed at the household level.

### 2.8 Variables

#### 2.8.1 Primary outcome measures

- Household secondary attack rate (SAR)
- COVID-19 positivity among household contacts

#### 2.8.2 Secondary outcome measures

- COVID-19 vaccination status
- Occurrence of new infection or reinfection
- Development of post-COVID symptoms
- Survival status

#### 2.8.3 Independent variables

- Sociodemographic characteristics of index cases and household contacts
- Clinical findings and disease severity
- Laboratory and imaging findings
- Household preventive measures (home isolation, mask use)
- Household characteristics (number of household members, number of rooms, use of shared living spaces)
- Contact-related behaviours with the index case (sleeping in the same room/bed, sharing meals, use of shared toilet facilities)

### 2.9 Long-term follow-up

Participants were followed for up to 2 years following SARS-CoV-2 infection.

During long-term follow-up, the following outcomes were assessed:

- COVID-19 vaccination status
- Occurrence of new infection or reinfection
- Post-COVID symptoms, defined as symptoms persisting for at least 2 months
- Mortality

### 2.10 Ethical approval

Ethical approval for the study was obtained from the Zonguldak Health Sciences Clinical Research Ethics Committee. All participants were informed about the study, and written and/or verbal informed consent was obtained. Personal data were protected in accordance with principles of confidentiality.

### 2.11 Statistical analysis

Statistical analyses were performed using IBM SPSS Statistics for Windows, version 23.0 (IBM Corp., Armonk, NY, United States). Because the primary outcome of interest was intra-household transmission, the household was defined as the unit of analysis. Accordingly, each household was treated as a single observational unit in analyses evaluating the occurrence of secondary transmission, thereby accounting for clustering of individuals within the same household and avoiding the assumption of independence between household members.

Categorical variables were summarised as frequencies and percentages, whereas continuous variables were presented as mean ± standard deviation (SD) or median (minimum–maximum), depending on data distribution. Normality of continuous variables was assessed using the Kolmogorov–Smirnov test.

Comparisons of continuous variables between groups were performed using the Mann–Whitney U test, while categorical variables were analysed using the chi-square test. Factors potentially associated with intra-household transmission (defined as the occurrence of at least one secondary SARS-CoV-2 case within the household following the index case) were initially evaluated using univariable analyses.

Variables with a *p*-value <0.20 in univariable analyses, together with variables considered clinically or epidemiologically relevant based on prior literature, were subsequently included in a multivariable logistic regression model to identify independent predictors of intra-household transmission. Results were expressed as odds ratios (ORs) and adjusted odds ratios (aORs) with corresponding 95% confidence intervals (CIs).

Potential multicollinearity among independent variables was assessed prior to model construction. Model goodness-of-fit was evaluated using the Hosmer–Lemeshow test. A two-sided *p*-value <0.05 was considered statistically significant. Missing data were minimal and were handled using complete-case analysis.

## 3 Results

A total of 202 households were contacted and assessed for eligibility. Among these households, 83 households either declined participation or did not meet the inclusion criteria, while 119 households agreed to participate and were included in the study. These households comprised 501 individuals, all of whom completed the follow-up period, with no households lost during follow-up.

The minimum required sample size for the study was calculated as 100 households, and the inclusion of 119 households exceeded this threshold, ensuring adequate statistical reliability.

The participant recruitment process is presented in Figure 1.

The results are presented according to the unit of analysis and organised under the following headings: baseline descriptive characteristics of the study population, household-level secondary attack rate (SAR), COVID-19 positivity among household contacts, and long-term follow-up outcomes.

### 3.1 Baseline characteristics of the study population

A total of 501 individuals from 119 households were included in the study. Among all participants, 119 (23.7%) were identified as index cases and 150 (30.0%) were classified as secondary cases. Among the remaining household contacts, 92 individuals (18.3%) tested negative for SARS-CoV-2 by RT-PCR, while 140 individuals (28.0%) did not undergo diagnostic testing during the early phase of the pandemic, when testing was largely restricted to symptomatic cases.

Overall, 53.1% of participants were female and 42.6% were children aged under 18 years. Most households were located in urban areas (75.6%), and the mean household size was 4.22 ± 1.18 individuals. The main demographic and household characteristics of the study population are summarised in Table 1. Overall, intra-household transmission occurred in 75 of 119 households (63.0%). Household-level factors potentially associated with intra-household transmission were analysed using logistic regression, and the results are presented in Table 2.

**Table 1:** Baseline demographic and household characteristics of the study population.

Variable	*n* (%)/Mean ± SD
Number of households	119
Total number of individuals	501
COVID-19 status	
Index case	119 (23.7)
Secondary case	150 (30.0)
PCR-negative household contact	92 (18.3)
Untested individual	140 (28.0)
Sex	
Female	266 (53.1)
Male	235 (46.9)
Age group	
<18 years	213 (42.6)
≥18 years	288 (57.4)
Household characteristics	
Urban residence	90 (75.6)
Mean household size	4.22 ± 1.18
Households with ≥5 members	41 (34.5)
Households with ≥3 rooms	58 (48.7)
Presence of at least one individual with a chronic disease	188 (37.5)

This table presents the baseline demographic characteristics, COVID-19–related case distribution, and selected household characteristics of 501 individuals residing in 119 households included in the study. Approximately one quarter of participants were classified as index cases, while nearly one third were classified as secondary cases. More than half of the study population consisted of adult individuals, and the majority of households were located in urban areas. The mean household size was approximately four individuals, with one in three households comprising five or more members. Data are presented as *n* (%) or mean ± standard deviation (SD).

**Table 2:** Factors associated with intra-household transmission at the household level.

Variable	Category	*n* (households)	Intra-household transmission (%)	Univariable OR (95% CI)	*p*	Multivariable aOR (95% CI)	*p*
Index case age group	Child	38	52.6	Reference		Reference	
	Adult	81	69.1	2.02 (0.91–4.45)	0.083	2.24 (0.96–5.25)	0.063
Place of residence	Rural	29	58.6	Reference		Reference	
	Urban	90	65.6	1.34 (0.57–3.17)	0.500	1.80 (0.68–4.80)	0.239
Household size	<5 members	78	60.3	Reference		Reference	
	≥5 members	41	70.7	1.59 (0.71–3.59)	0.260	2.10 (0.85–5.15)	0.106
Number of rooms	>3 rooms	58	65.5	Reference		Reference	
	3 rooms	49	61.2	0.82 (0.39–1.76)	0.616	0.95 (0.40–2.25)	0.393
	<3 rooms	12	66.7	1.15 (0.32–4.06)	0.831	1.90 (0.44–8.32)	0.902
Shared meals with index case	No	18	50.0	Reference		Reference	
	Yes	101	66.3	1.97 (0.72–5.42)	0.189	2.05 (0.70–6.02)	0.191
Household mask use	No	65	67.7	Reference		Reference	
	Yes	54	59.3	0.69 (0.33–1.47)	0.341	0.63 (0.27–1.44)	0.270

Intra-household transmission was defined as the occurrence of at least one secondary COVID-19 case within the household following the index case. Univariable analyses were performed using single-variable logistic regression for each factor. Variables considered clinically and epidemiologically relevant were subsequently included in the multivariable logistic regression model. Results are presented as odds ratios (ORs) and adjusted odds ratios (aORs) with 95% confidence intervals (CIs). Reference categories correspond to rows left blank in the OR columns. A *p*-value <0.05 was considered statistically significant.

### 3.2 Household secondary attack rate

The mean household secondary attack rate (SAR) calculated across the 119 households was 40.7% ± 39.4%, with a median value of 33.0%. Associations between SAR and selected characteristics of the index case, including age, clinical features, and household isolation practices, were explored.

Household-level factors potentially associated with intra-household transmission were evaluated using logistic regression analysis, and the results are presented in Table 2.

Complete household infection, defined as infection of all household members within the household, was observed in 23.5% of households. In univariable analysis, mask use within the household was associated with a significantly reduced likelihood of complete household infection (OR: 0.31; 95% CI: 0.12–0.81; *p* = 0.016). This association remained statistically significant in multivariable analysis (aOR: 0.34; 95% CI: 0.12–0.92; *p* = 0.034). No other index case– or household-level characteristics were independently associated with complete household infection (Table 3).

**Table 3:** Factors associated with complete household infection (all members infected).

Variable	Category	*n* (households)	Complete household infection (%)	Univariable OR (95% CI)	*p*	Multivariable aOR (95% CI)	*p*
Index case age group	Child	38	15.8	Reference		Reference	
	Adult	81	27.2	1.99 (0.73–5.41)	0.178	2.18 (0.76–6.26)	0.148
Place of residence	Rural	29	24.1	Reference		Reference	
	Urban	90	23.3	0.96 (0.36–2.55)	0.929	1.15 (0.38–3.55)	0.804
Household size	<5 members	78	25.6	Reference		Reference	
	≥5 members	41	19.5	0.70 (0.28–1.77)	0.455	0.93 (0.34–2.57)	0.885
Number of rooms	>3 rooms	58	19.0	Reference		Reference	
	3 rooms	49	28.6	1.60 (0.68–3.75)	0.280	1.60 (0.61–4.21)	0.337
	<3 rooms	12	25.0	1.09 (0.27–4.35)	0.899	2.05 (0.39–10.91)	0.400
Household mask use	No	65	32.3	Reference		Reference	
	Yes	54	13.0	0.31 (0.12–0.81)	0.016	0.34 (0.12–0.92)	0.034

Complete household infection was defined as SARS-CoV-2 infection occurring in all individuals residing within the household. Univariable analyses were performed using binary logistic regression for each variable. Multivariable analysis was conducted by including variables considered clinically and epidemiologically relevant. Odds ratios (ORs) and adjusted odds ratios (aORs) are presented with 95% confidence intervals (CIs). Reference categories correspond to rows left blank in the OR columns. A *p*-value <0.05 was considered statistically significant.

### 3.3 COVID-19 positivity among household contacts and long-term follow-up outcomes

Among household contacts, excluding index cases, contact-related characteristics associated with COVID-19 positivity were evaluated. In addition, long-term follow-up analyses assessed the occurrence of COVID-19 during subsequent epidemic waves, the presence of post-COVID symptoms, and mortality outcomes within the study population. COVID-19 positivity among household contacts and long-term follow-up findings are summarised in Table 4.

**Table 4:** COVID-19 positivity among household contacts and long-term follow-up outcomes.

Variable	COVID-19 positive *n*/*N* (%)	*p*
Contact-related characteristics		
Sleeping in the same room	57/107 (53.3)	0.001
Sleeping in the same bed	42/74 (56.8)	0.001
Sharing meals	138/333 (41.4)	0.035
Age group		
<1 year	7/9 (77.8)	0.013
Long-term follow-up		
Reinfection (entire cohort)	42/501 (8.4)	–
Post-COVID symptoms (confirmed cases)	73/269 (27.1)	–
Mortality	3/269 (1.1)	–

This table jointly presents selected contact-related characteristics associated with laboratory-confirmed COVID-19 positivity among household contacts, excluding the index case, as well as long-term follow-up outcomes of the study population. Associations between close-contact behaviours (sleeping in the same room or bed, sharing meals) and COVID-19 positivity are reported as *n*/*N* (%), where N refers to the number of household contacts at risk. Reinfection is reported for the entire study cohort (*N* = 501), whereas post-COVID symptoms and mortality are reported among confirmed COVID-19 cases (*N* = 269). In addition, the table summarises the occurrence of COVID-19 during subsequent epidemic waves, the development of post-COVID symptoms, and mortality rates observed during the follow-up period.

## 4 Discussion

In this ambispective, community-based study with long-term follow-up, we demonstrated that intra-household transmission of SARS-CoV-2 is high in households including children, and that transmission dynamics are shaped not only by the clinical characteristics of index cases but also strongly influenced by daily living practices and intra-household contact behaviours. The household secondary attack rate exceeding 40% observed in our study highlights the critical role of households as focal points for SARS-CoV-2 transmission during the early phase of the pandemic, under pre-vaccination conditions and in real-world settings where isolation measures were limited.

Our study provides a novel and comprehensive contribution to the literature by evaluating determinants of transmission in paediatric-inclusive households beyond short-term infection risk, incorporating long-term outcomes such as reinfection, post-COVID symptoms, and mortality over an approximately two-year follow-up period.

Household transmission studies are of central importance for understanding the dynamics of respiratory virus spread, particularly for highly transmissible pathogens such as SARS-CoV-2, where close and prolonged intra-household contacts play a fundamental epidemiological role. Households are recognised as high-risk settings for SARS-CoV-2 transmission due to extended contact duration, enclosed environments, and shared living spaces, and a substantial proportion of COVID-19 cases have been reported to occur in family clusters (5–7).

In this study, the mean household secondary attack rate (SAR) was found to be 40.7%, which is higher than the average SAR values ranging between 15 and 30% reported in the literature from different countries (8–10). The high SAR observed in our study may be attributable to early pandemic conditions, the pre-vaccination period, the extended follow-up duration, and limited implementation of isolation measures within households (11–14). Furthermore, during the period in which the study was conducted in our country, testing strategies predominantly focused on symptomatic individuals, which may have allowed the true extent of intra-household transmission to be captured only through prolonged follow-up (11, 14, 15).

During the study period, the wild-type strain and the Alpha variant, which were predominant in the country, were reported to have higher transmissibility than previously circulating respiratory pathogens, but lower transmissibility compared with the Delta and Omicron variants (16–18). Nevertheless, the observation of high SAR values in our study suggests that household contact intensity and behavioural factors, rather than variant-specific characteristics, may have played a more decisive role. Adult age of the index case, the presence of cardiovascular comorbidities, and symptoms such as cough, olfactory dysfunction, and fatigue/weakness were associated with higher odds of intra-household transmission. These findings are consistent with the existing literature suggesting higher viral loads and increased aerosol and droplet shedding in symptomatic individuals (14, 19–21). The lower SAR observed in households with asymptomatic index cases may be explained by reduced aerosol generation; however, it should also be considered that asymptomatic individuals may play a substantial role in silent transmission due to lower disease awareness (3, 22, 23).

When intra-household contact behaviours were examined, all close-contact practices involving the index case—such as sleeping in the same room or bed and sharing meals—were found to be significantly associated with COVID-19 positivity. In particular, bed-sharing emerged as the highest-risk form of contact for intra-household transmission. These findings are consistent with previous studies demonstrating that close and prolonged contact is a key determinant of SARS-CoV-2 transmission (14). In contrast, implementation of household isolation measures was associated with a significant reduction in the household secondary attack rate, supporting isolation as one of the most effective and feasible strategies for preventing intra-household transmission (19, 24).

When household contacts were analysed according to age groups, a notably high rate of COVID-19 positivity was observed among children under 1 year of age. This finding may be related to the inevitability of close contact in this age group, the inability to use masks, and high caregiving needs. The similar overall positivity rates observed between paediatric and adult contacts suggest that the extended follow-up duration and broad contact definitions employed in this study may have enabled more accurate detection of infection among children (13, 25).

During long-term follow-up, the reinfection rate was found to be 8.4%, which is higher than that reported in some published series. This finding may be attributable to the extended follow-up duration and the inclusion of periods during which the Delta and Omicron variants were predominant (26, 27).

Post-COVID symptoms were identified in 27.1% of confirmed cases, with fatigue/weakness and chest pain being the most frequently reported symptoms. These findings are consistent with the existing literature indicating that the prevalence of post-COVID manifestations has been reported across a wide range and is influenced by heterogeneity in study populations (28–30). The higher frequency of post-COVID manifestations observed among females and in the adult age group suggests that both biological and psychosocial factors may play a role (28–31).

The lower occurrence of post-COVID symptoms among vaccinated individuals supports the possibility that COVID-19 vaccines may have a protective effect against long-term symptoms (32, 33).

In our study, the COVID-19–related mortality rate was 1.1%, with all deaths occurring among older individuals with multiple comorbidities. This finding is consistent with previous studies highlighting the decisive role of advanced age and underlying conditions in COVID-19 mortality (34–36).

This study has several limitations that should be considered when interpreting the findings. First, the single-centre design may limit the generalisability of the results to other populations and settings. Second, during the early phase of the COVID-19 pandemic, testing capacity was limited in Türkiye and routine RT-PCR testing of asymptomatic household contacts was not systematically implemented. Consequently, some asymptomatic individuals were not tested and were analysed together with PCR-negative participants in the transmission analyses. This approach reflects real-world testing practices during the early pandemic period but may have introduced potential outcome misclassification and may have led to underestimation of the true household secondary attack rate. However, the primary objective of this study was to identify household-level determinants of intra-household transmission rather than to estimate the precise magnitude of the secondary attack rate. Accordingly, the principal regression analyses were conducted at the household level, defining transmission as the occurrence of at least one secondary case within the household. Because the analytical unit was the household rather than the individual contact, potential misclassification at the individual contact level is unlikely to substantially influence the main conclusions regarding factors associated with transmission. Third, field access constraints related to pandemic conditions and the identification of households through index cases and contact tracing pathways may have introduced a degree of selection bias. Finally, information regarding intra-household contact behaviours and preventive measures was obtained through participant self-report, which may be subject to recall bias.

Despite these limitations, the household-based design of the study, its focus on families including children, and the extended follow-up period provide valuable insights into intra-household SARS-CoV-2 transmission dynamics and long-term outcomes in a real-world setting.

## 5 Conclusion

In conclusion, this ambispective, community-based study with long-term follow-up demonstrates that intra-household transmission of SARS-CoV-2 is high in households including children, and that transmission risk is largely determined by the clinical characteristics of index cases as well as intra-household contact behaviours. In particular, daily living practices involving close and prolonged contact emerge as key determinants of household transmission.

Data obtained over an approximately two-year follow-up period indicate that intra-household transmission should not be viewed solely in terms of acute infection risk, but rather evaluated alongside long-term outcomes such as reinfection, post-COVID symptoms, and mortality. The increased susceptibility observed among highly care-dependent groups, such as children under 1 year of age, underscores the need for careful consideration of the role of paediatric populations within household transmission chains.

The demonstrated effectiveness of household isolation measures in significantly reducing the secondary attack rate supports the critical importance of household-targeted preventive strategies from a public health perspective. These findings may inform future responses to emerging respiratory epidemics by prioritising households with children as key intervention settings and by promoting the development of household-based, behavior-focused prevention strategies.

## Data Availability

The raw data supporting the conclusions of this article will be made available by the authors, without undue reservation.
